# Early Detection of Hemodynamic Responses Using EEG: A Hybrid EEG-fNIRS Study

**DOI:** 10.3389/fnhum.2018.00479

**Published:** 2018-11-29

**Authors:** M. Jawad Khan, Usman Ghafoor, Keum-Shik Hong

**Affiliations:** ^1^School of Mechanical Engineering, Pusan National University, Busan, South Korea; ^2^School of Mechanical and Manufacturing Engineering, National University of Science and Technology, Islamabad, Pakistan; ^3^Department of Cogno-Mechatronics Engineering, Pusan National University, Busan, South Korea

**Keywords:** brain-computer interface (BCI), functional near-infrared spectroscopy (fNIRS), electroencephalography (EEG), hybrid EEG-fNIRS, hemodynamic response, vector phase diagram, classifier

## Abstract

Enhanced classification accuracy and a sufficient number of commands are highly demanding in brain computer interfaces (BCIs). For a successful BCI, early detection of brain commands in time is essential. In this paper, we propose a novel classifier using a modified vector phase diagram and the power of electroencephalography (EEG) signal for early prediction of hemodynamic responses. EEG and functional near-infrared spectroscopy (fNIRS) signals for a motor task (thumb tapping) were obtained concurrently. Upon the resting state threshold circle in the vector phase diagram that uses the maximum values of oxy- and deoxy-hemoglobin (Δ*HbO* and Δ*HbR*) during the resting state, we introduce a secondary (inner) threshold circle using the Δ*HbO* and Δ*HbR* magnitudes during the time window of 1 s where an EEG activity is noticeable. If the trajectory of Δ*HbO* and Δ*HbR* touches the resting state threshold circle after passing through the inner circle, this indicates that Δ*HbO* was increasing and Δ*HbR* was decreasing (i.e., the start of a hemodynamic response). It takes about 0.5 s for an fNIRS signal to cross the resting state threshold circle after crossing the EEG-based circle. Thus, an fNIRS-based BCI command can be generated in 1.5 s. We achieved an improved accuracy of 86.0% using the proposed method in comparison with the 63.8% accuracy obtained using linear discriminant analysis in a window of 0~1.5 s. Moreover, the active brain locations (identified using the proposed scheme) were spatially specific when a *t-*map was made after 10 s of stimulation. These results demonstrate the possibility of enhancing the classification accuracy for a brain-computer interface with a time window of 1.5 s using the proposed method.

## Introduction

In order to reduce the brain signal detection time and to improve the classification accuracy for brain-computer interfaces (BCIs), concurrent measurement of brain commands using electroencephalography (EEG), and functional near-infrared spectroscopy (fNIRS) at a focused local brain region is proposed. This paper presents a novel hybrid technique for the early detection of fNIRS signals based upon the power spectra of EEG signals to conclude the occurrence of hemodynamic responses. Over the past decades, computer and communication technologies have developed rapidly. BCI techniques have become an indispensable tool for patients' daily life. The goal of BCI is to make patients' life more convenient and natural in daily living environment (Ding et al., 2017). The primary goal of BCI is to assist patients (typically, in locked-in state) to interact with the living environment using only brain signals (Coyle et al., 2003; Nicolas-Alonso and Gomez-Gil, 2012). It is important for patients to control an external device easily, accurately, quickly, and with a sufficient number of commands (e.g., robots, wheelchairs, etc.) (Turnip et al., 2011; Hong et al., 2018a,b). However, if we increase the number of commands, the accuracy drops in most BCIs. In order to compensate for the reduction in accuracy and also to improve the brain signal detection time, the concept of hybridization has been introduced (Pfurtscheller et al., 2010).

A hybrid EEG-fNIRS BCI has great potential to enhance the classification accuracy and to increase the number of commands than regular BCI systems (Hong and Khan, 2017). The early hybrid EEG-fNIRS BCI developed in Fazli et al. (2012) already showed that a simultaneous decoding of EEG signals along with oxy- and deoxy-hemoglobin (Δ*HbO* and Δ*HbR*) signals acquired using fNIRS can enhance the classification accuracy. The hybrid method discussed in Tomita et al. (2014) demonstrated that the accuracy for a motor task was improved by combining the probability scores obtained from EEG and fNIRS classification using a joint classifier. Many other studies also demonstrated that BCI accuracy can be enhanced by decoding EEG and fNIRS features simultaneously (Putze et al., 2014; Koo et al., 2015; Yin et al., 2015; Buccino et al., 2016). The studies of Khan et al. (2014) and Khan and Hong (2017) also showed that the decoding of EEG and fNIRS signals from different brain regions can be adopted to increase the number of commands without sacrificing the classification accuracy.

Aside from the issues of enhancing the classification accuracy and increasing the number of commands, results on optimal feature selection in hybrid EEG and fNIRS frameworks and a unified classification model for hybrid systems are still insufficient in the literature (Keles et al., 2016; Park et al., 2016; Hong and Khan, 2017). The bottleneck in the hybrid EEG-fNIRS framework is still considered to be the command generation time due to the inherent delay in hemodynamic signals of fNIRS. Therefore, in this paper, a quick detection method of the occurrence of hemodynamic responses is investigated.

Several researchers have worked on the problem of finding an optimal window size for BCI. However, the literature yet did not show a conclusive work on a standardized window selection method for simultaneous decoding of brain activities. In the work of Tomita et al. (2014), a window size of 10 s was reported for simultaneous EEG-fNIRS feature extraction, but it results in a 10 s delay which is inappropriate for controlling an external device. For hybrid EEG-fNIRS, various window sizes for feature extraction and classification have been reported in the literature (Blokland et al., 2014; Fazli et al., 2015; Yin et al., 2015; Buccino et al., 2016). In most cases, a window size larger than 5 s was used for feature extraction and classification for hybrid EEG-fNIRS BCI. This will cause an unnecessary delay in making the final decision because the data should be processed using the data during the window.

However, the window size for command generation can be reduced by either initial dip detection (Jasdzewski et al., 2003; Yoshino and Kato, 2012; Naseer and Hong, 2015) or fast optical response (Hu et al., 2011) methods instead of relying on the hemodynamic responses. Recent fNIRS-BCI studies have shown the feasibility of using initial dips for BCI application, which generate multiple commands from the prefrontal cortex using initial dip features within a 2.5 s window (Hong and Naseer, 2016; Zafar and Hong, 2017). A recent hybrid EEG-fNIRS study also combined EEG and fNIRS (initial dip) features in a 0~2 s window to enhance the accuracy of a BCI system. However, the command generation time needs to be further reduced for practical applications (Li et al., 2017) by extracting differentiable features (Kim et al., 2016; Naseer et al., 2016a; Huang et al., 2017; Song et al., 2018), or by using signal processing algorithms (Santosa et al., 2013; Gui et al., 2017; Hamadache and Lee, 2017; Yaqub et al., 2018), or by application of better classification approach (Bui et al., 2016; Choi et al., 2016; Naseer et al., 2016b; Azizi et al., 2017). Therefore, a decision making scheme that can detect fNIRS signals in a window smaller than 2 s should be the primary focus of the current hybrid EEG-fNIRS research. To the best of the authors' knowledge, an algorithm that can simultaneously extract and classify features from the same brain area in <2 s in an EEG-fNIRS system has not been developed yet.

The main objective of this paper is to develop a systematic way that EEG signals are combined with fNIRS signals for the purpose of improving the detection time without knowing the start time of a brain task. The task adopted in this paper is thumb movement (not a cognitive task). As EEG and fNIRS signals caused by thumb movement can be measured in most subjects, a small area (4 × 5 cm) in the motor cortex focusing on C3 in the International 10–20 System was focused. For the proposed idea on a motor task, the consistency of a particular subject in validating the proposed signal processing scheme is more crucial than investigating an averaged value among many subjects. The hypotheses of this study are (i) EEG signals can be used in detecting/predicting the hemodynamics response of fNIRS, and (ii) a false detection of brain activity using EEG can be reduced by fNIRS at a delayed hemodynamic response time.

## Methods

### Participants

Since the activity for brain signal acquisition is a finger movement task (i.e., right thumb finger) that can be performed by most people without difficulty, no particular attention has been given to the subjects' age, gender, and the previous experience on BCI (except consistency). Three male subjects (age: mean 28.5 ± 2.5 years, hairstyle: very short hair) participated in the experiment to validate a new signal processing scheme. All three subjects were healthy, right handed, and had normal or corrected-to-normal vision, and none had a history of any neurological or visual disorder. All were given a detailed description on the experimental procedure prior to the beginning of experiment, and all provided written consent after having been informed. The experiment was conducted in accordance with the latest Declaration of Helsinki upon the approval of the Pusan National University Institutional Review Board.

### Channel configuration and signal processing

**Figure 1:**
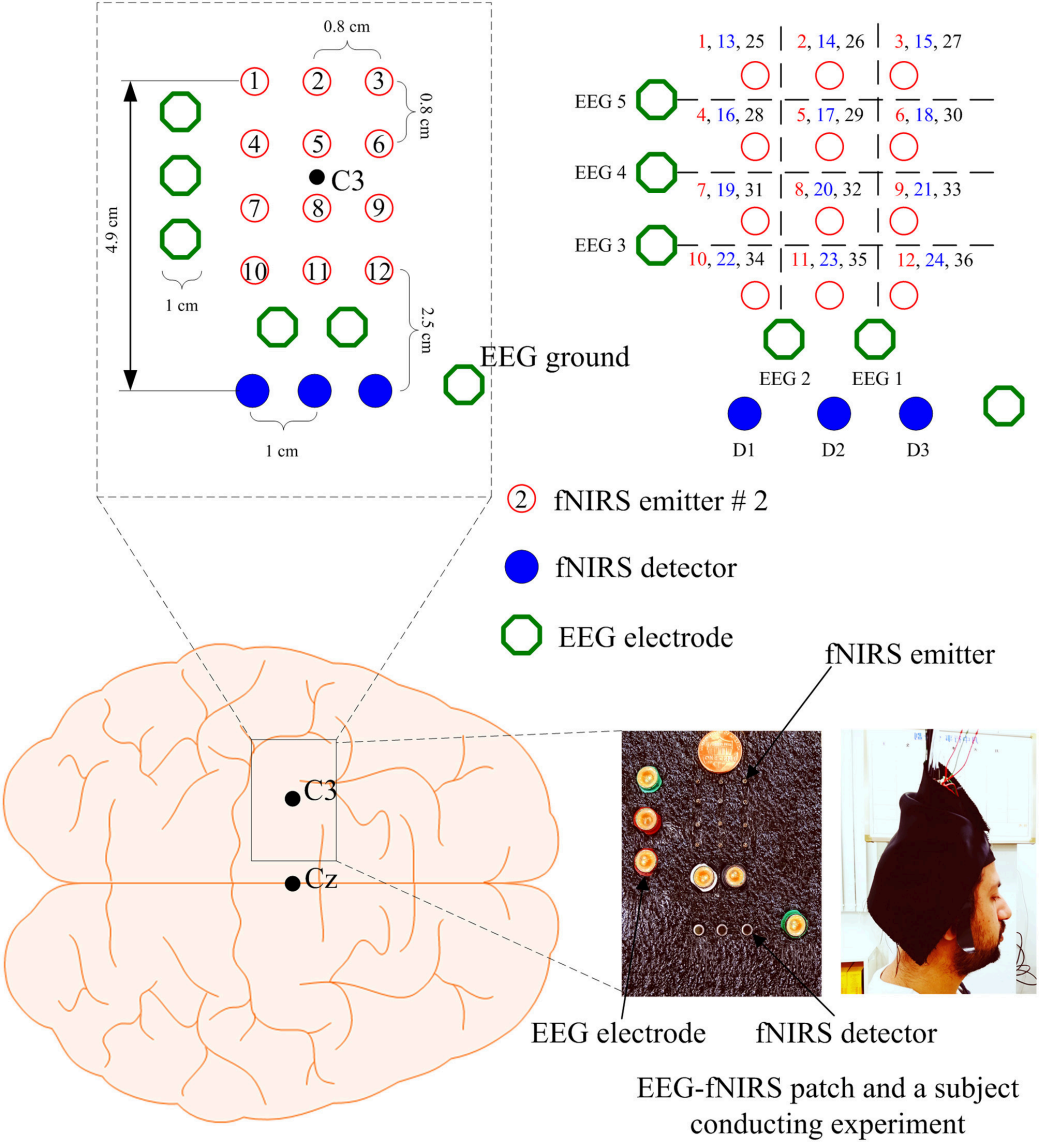
Configuration of EEG electrodes and fNIRS optodes focusing on C3 reference point in the left motor cortex, and photos of EEG-fNIRS patch and a subject performing experiment: the numbers in the top right corner indicate the fNIRS channel numbers.

Brain signals generated by tapping of right thumb were acquired at a sampling rate of 9.19 Hz from the left motor cortex using a frequency-domain fNIRS system (ISS Imagent, ISS Inc., USA). The system utilizes near-infrared light of two wavelengths (690 and 830 nm). In this study, 3 detectors and 12 emitters were used in a dense emitter-detector configuration, see Figure 1, to examine the C3 area on the left motor cortex (Nguyen and Hong, 2016; Nguyen et al., 2016; Zafar and Hong, 2018). In accordance with the International 10–20 System, the detectors were positioned surrounding the C3 point as a reference point. The standard EEG cap (Neuroelectrics® Neoprene Headcap, Barcelona, Spain) was used to access the C3 location for individual subjects. Also, five Ag/AgCl EEG electrodes were placed around/beside the fNIRS optodes. The EEG data were recorded using a g-MOBIlab+ biosignal acquisition device (Christoph Guger, Austria) at a sampling rate of 256 Hz. In Figure 1, the hollow circle with a number in it denotes an fNIRS emitter (total 12 emitters), the (blue) filled circles (D1, D2, D3) represent the fNIRS detectors (total 3 detectors), and the hollow octagons indicate the EEG electrodes (3 vertically, 2 horizontally). Thirty-six channels were configured using emitter-detector combinations (see the top right corner in Figure 1). The numbers in the top-right corner denote the fNIRS channels: For instance, emitter 1 and detector 1 makes Ch. 1, emitter 2 and detector 1 makes Ch. 2, emitter 1 and detector 2 makes Ch. 13, etc. As seen from the left photo (hybrid EEG-fNIRS patch) and the channel configuration, EEG electrodes #1 and #2 were placed on the top of fNIRS channels, and EEG electrodes #3, #4, and #5 were located to the left side of the fNIRS emitters.

ISS Imagent data acquisition and analysis software (ISS-Boxy) were used to obtain the raw intensity data. The intensity data were then converted to Δ*HbO* and Δ*HbR* using the ISS-Boxy software, with extinction coefficients ε_HbO_ = 0.95 mM^−1^cm^−1^ and ε_HbR_ = 4.93 mM^−1^cm^−1^ for the 690 nm wavelength and ε_HbO_ = 2.135 mM^−1^cm^−1^ and ε_HbR_ = 1.791 mM^−1^cm^−1^ for the 830 nm wavelength, according to the modified Beer-Lambert law (MBLL) (Baker et al., 2014; Bhatt et al., 2016). The converted data of Δ*HbO* and Δ*HbR* were pre-processed to remove the physiological noises related to the respiration, cardiac activity, and low-frequency drift signals. For this, fourth-order Butterworth low- and high-pass filters with cutoff frequencies of 0.15 Hz and 0.01 Hz, respectively, were used to filter off the noises caused by respiration, cardiac activity, and low-frequency drift fluctuations from the converted hemodynamic signals (Hong et al., 2015; Khan and Hong, 2015; Weyand et al., 2015; Hong and Santosa, 2016). In this study, any motion-artifact correction algorithm was not used because any motion artifacts were not found from the raw data. It is noted that all three subjects have had a number of previous experiences in performing fNIRS experiments. The band-pass filtering of the EEG electrode signals into the α*-*, β*-*, Δ*-*, and θ*-*bands (acquired via 8–12, 12–28, 0.5–4, and 4–8 Hz, respectively) allowed the isolation of β*-*band, which corresponds to the motor activity (Lotte et al., 2007; Ortiz-Rosario and Adeli, 2013; Ramadan and Vasilakos, 2017).

### Experimental paradigm

**Figure 2:**
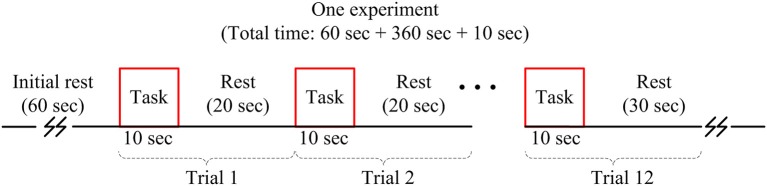
Experimental paradigm used for acquiring brain signals.

A tapping of right thumb task associated with the left motor cortex was investigated. The subjects were seated on a comfortable chair and were instructed to avoid any body movement, particularly the head, as much as possible during the experiment. The experiment was conducted in a dark and quiet room to avoid any interference from the environment. Figure 2 shows the experimental paradigm used in this study. One experiment consists of 12 trials of thumb tapping task with pre- and post-rest periods of 60 and 10 s, respectively. The duration of one trial was 30 s, which includes a 10 s activity task followed by a rest period of 20 s. During the task period, the subjects were instructed to tap their right thumb as fast as they could, without paying attention to the number of taps. A computer screen indicating thumb finger taps during the task period was placed in front of the subject. During the rest period, a black screen was shown. The subjects were also instructed to keep their eyes open during the experiment. In conclusion, the total number of data in this work is 1,296 (i.e., 36 channels × 12 trials × 3 subjects).

### Cross-checking strategy

**Figure 3:**
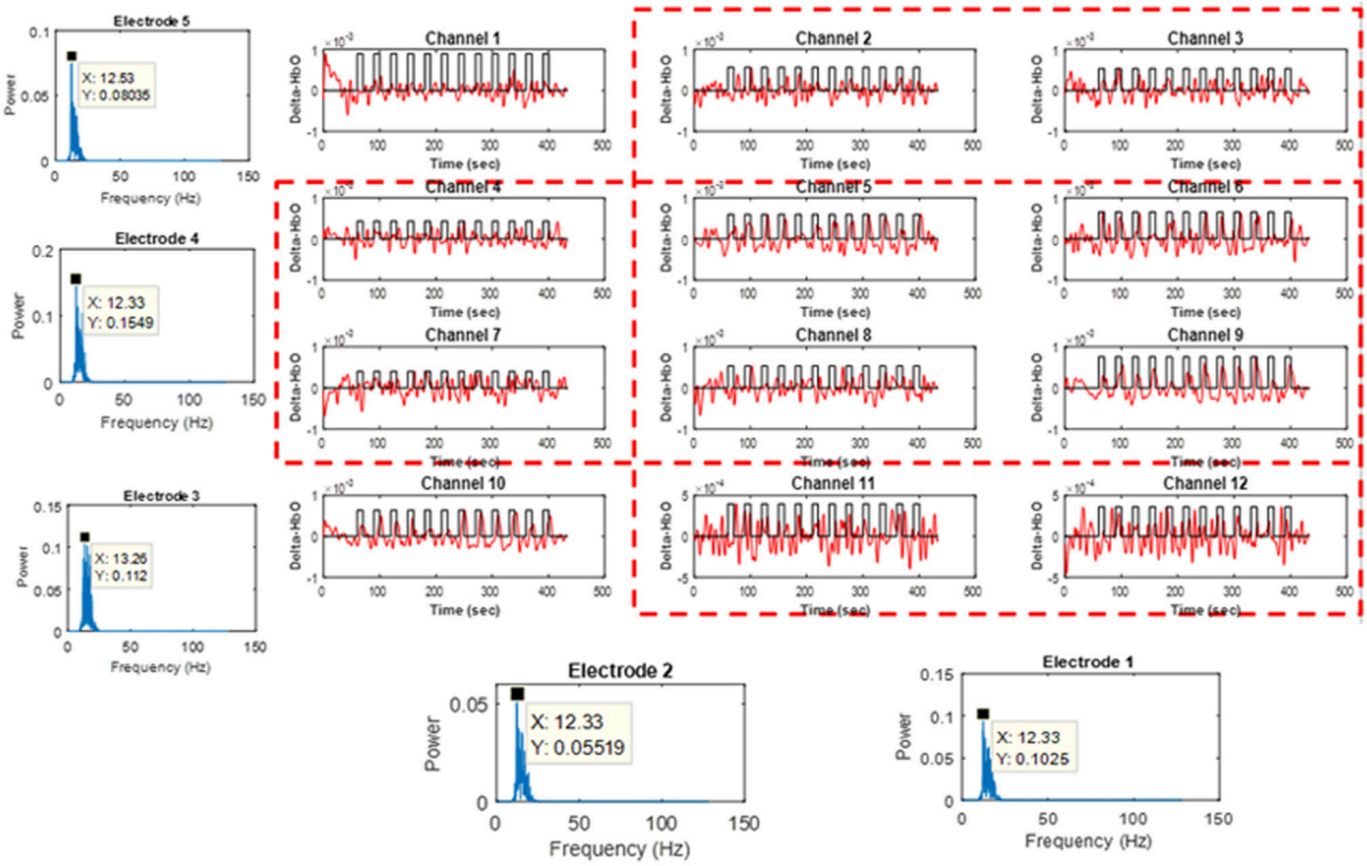
Cross-checking strategy for finding active fNIRS channels using EEG electrodes showing the highest power for 1 s, in which EEG electrodes are placed vertically and horizontally (Sub. 1, fNIRS channels 13–36 are omitted).

Figure 3 shows the perpendicular arrangement of 5 EEG electrodes (3 vertically, 2 horizontally), and 12 plots of the hemodynamic responses of 12 fNIRS channels (the plots of 13–36 channels are not shown). Upon the tapping task, the power of individual EEG electrodes were measured. Assume that EEG electrode #4 among the vertically arranged electrodes showed the highest power and EEG electrode #1 among the horizontally placed ones showed the highest power. Then, as far as fNIRS channel selection is concerned, channels 5, 6, 8, and 9 can be examined because they are nearest ones to the two EEG electrodes (Joundi et al., 2012; Solis-Escalante et al., 2012; Wagner et al., 2016). Table 1 summarizes the selected EEG electrodes and fNIRS channels per subject. For instance, for Subject 1, since EEG channels 1 and 4 showed the highest power, fNIRS channels 5, 6, 8, 9, 17, 18, 20, 21, 29, 30, 32, 33 were pre-selected by using the strategy in Figure 3. The problem in this method however is that twelve channels are always selected, which contain information from both active and inactive channels. Thus, a precise determination of the most activated channels is not possible. Therefore, a further reduction of channels is required to correctly identify the activated brain region. Now, we shifted our approach to vector-phase analysis.

**Table 1:** Selected EEG and fNIRS channels per subject.

**Subject**	**EEG**	**EEG + vector phase analysis**	**fNIRS channels selected by the cross-checking strategy in Figure 3**
1	1 and 4	Chs. 17, 18, 29, and 33	5, 6, 8, 9, 17, 18, 20, 21, 29, 30, 32, 33
2	1 and 3	Chs. 32, and 33	8, 9, 11, 12, 20, 21, 23, 24, 32, 33, 35, 36
3	1 and 4	Chs. 17, 21, and 33	5, 6, 8, 9, 17, 18, 20, 21, 29, 30, 32, 33

### Vector-phase analysis

Vector-phase analysis is a systematic method that can trace the entire hemodynamic response using two components (i.e., HbO and HbR) in the ΔHbO-ΔHbR phase diagram. An initial dip is the early decrease/increase in Δ*HbO*/Δ*HbR* as result of neurovascular coupling. Therefore, both the initial dip as well as the hemodynamic signal can be detected using the vector-phase diagram (Hong and Zafar, 2018). The original method uses eight phases that are created using a pair of Δ*HbO* and Δ*HbR* signals (or Δ*HbT* and Δ*COE* signals) (Yoshino and Kato, 2012; Sano et al., 2013; Yoshino et al., 2013; Oka et al., 2015). The vector components Δ*HbT* (total hemoglobin) and Δ*COE* (cerebral oxygen exchange) are obtained by rotating the vector coordinate system defined by Δ*HbO* and Δ*HbR* by 45° counterclockwise using the following equations:

(1) $$\begin{array}{l} \Delta HbT=\frac{1}{\sqrt{2}}\left(\Delta HbO+\Delta HbR\right), \end{array}$$

(2) $$\begin{array}{l} \Delta COE=\frac{1}{\sqrt{2}}\left(\Delta HbR-\Delta HbO\right). \end{array}$$

The magnitude and phase of a vector *p* = (Δ*HbO*, Δ*HbR*) in this plane can be calculated as

(3) $$\begin{array}{l} |p|=\sqrt{\Delta HbO^{2}+\Delta HbR^{2}}, \end{array}$$

(4) $$\begin{array}{l} ∠p=\tan^{-1}\left(\frac{\;\Delta \;HbR}{\;\Delta \;HbO}\right)=\tan^{-1}\left(\frac{\;\Delta \;COE}{\;\Delta \;HbT}\right)+45^{o}. \end{array}$$

The phase diagram is divided into eight phases/regions according to four components (Δ*HbO*, Δ*HbR*, Δ*HbT*, and Δ*COE*). The details of the vector diagram are summarized in Table 2.

**Table 2:** Different phases in the vector diagram related to initial dip and hemodynamics.

**Phase**	**1**	**2**	**3**	**4**	**5**	**6**	**7**	**8**
ΔHbO	Positive	Positive	Negative	Negative	Negative	Negative	Positive	Positive
ΔHbR	Positive	Positive	Positive	Positive	Negative	Negative	Negative	Negative
ΔHbT	Positive	Positive	Positive	Negative	Negative	Positive	Negative	Positive
ΔCOE	Negative	Positive	Positive	Positive	Positive	Negative	Negative	Negative
Condition	ΔHbO > ΔHbR	ΔHbO < ΔHbRΔHbT > ΔCOE	ΔHbT < ΔCOE		ΔHbO < ΔHbR	ΔHbO > ΔHbR	ΔHbT > ΔCOE	ΔHbT < ΔCOE
Type	Initial dip					Hemodynamic		

### Modified vector-phase analysis with a second circle based on EEG activated window

A threshold circle (the red solid circle) based on the maximum magnitude during the resting state was used for the detection of hemodynamic responses in the previous studies (Hong and Naseer, 2016; Zafar and Hong, 2017). The circle was placed based on the highest values of Δ*HbO* and Δ*HbR* in the resting state. As for the criterion used, an initial dip appears if the magnitude *p* crosses the threshold circle in Phases 3, 4, and 5. If there were no threshold circle, a resting state fluctuation with Δ*COE* > 0 could easily be interpreted as an initial dip. The radius of the resting state threshold circle R_1_ is defined as follows:

(5) $$\begin{array}{l} {\text{R}}_{1}=\max{\left(\Delta HbO_{resting}^{2}+\Delta HbR_{resting}^{2}\right)}^{1/2}. \end{array}$$

**Figure 4:**
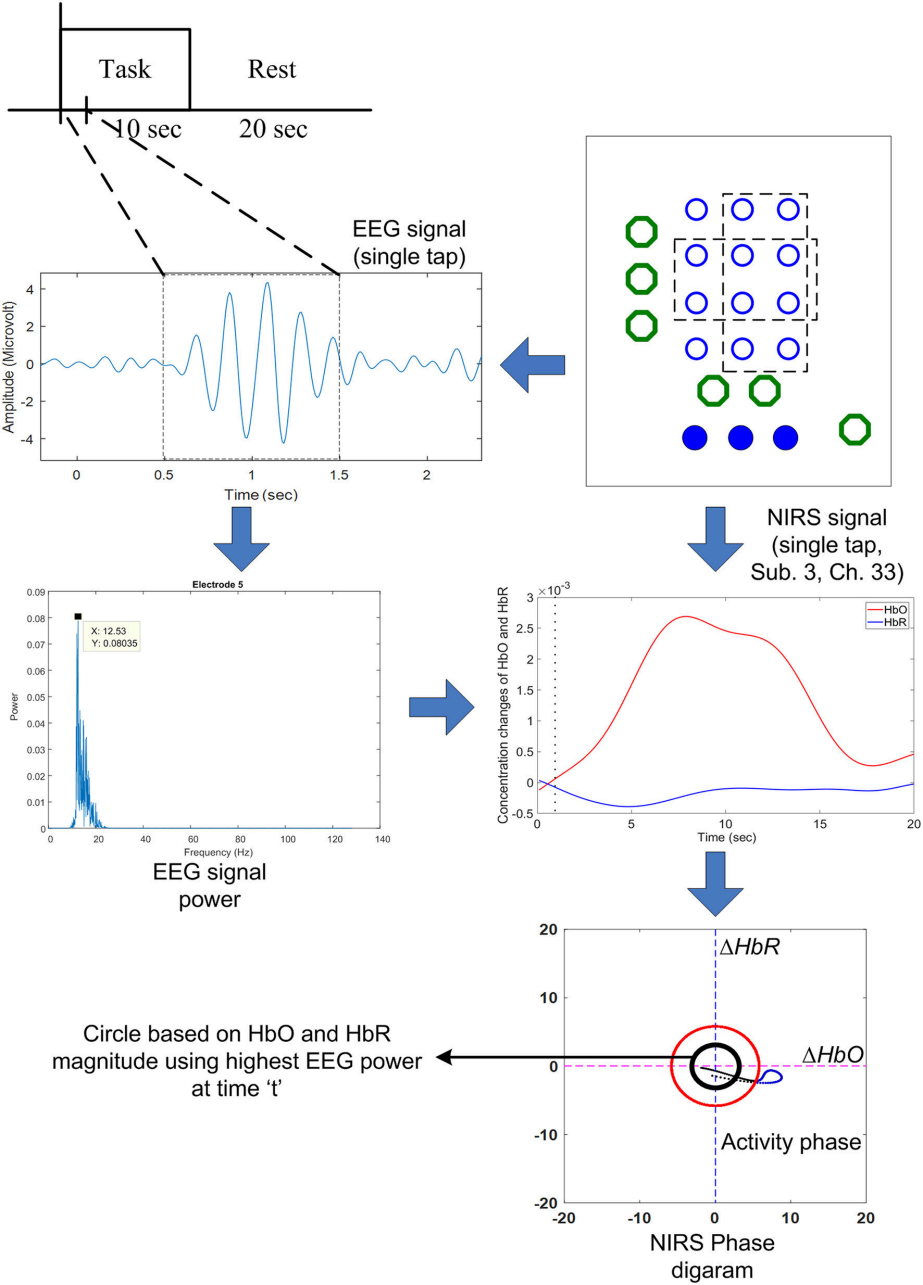
The proposed scheme to predict the early hemodynamic response using the highest EEG power obtained from the 1 s time window: If the EEG power is over a certain threshold value, the (black) threshold circle is drawn using Equation (6).

For a conventional hemodynamic signal, Δ*HbO* increases and Δ*HbR* decreases after a task is performed. Therefore, Phases 7 and 8 correspond to the hemodynamic signals in the vector diagram. The problem with the previous method is that a false negative increase in the hemodynamic signal can cause *p* to cross the threshold circle made by the baseline period, which might be deemed as a positive detection as per the criterion used. To solve the problem of false detection of hemodynamic signals, we propose a new method that involves adding a second circle based on the Δ*HbO* and Δ*HbR* values, which correspond to the window in which EEG sensing is active. To reduce the computation time, we only used four quadrants instead of the phases in the vector diagram. The only quadrant in which Δ*HbO* is positive and Δ*HbR* is negative is the fourth quadrant. The trajectory of vector *p* in the fourth quadrant from the smaller circle to the larger circle indicates that Δ*HbO* is increasing and Δ*HbR* is decreasing. The radius R_2_ of the smaller circle is given as:

(6) $$\begin{array}{c} {\text{R}}_{2}={\left(\max{\left(\Delta HbO\right)}_{w}^{2}+\max{\left(\Delta HbR\right)}_{w}^{2}\right)}^{1/2}, \end{array}$$

where *w* is the window number that corresponds to EEG activation. Figure 4 shows the proposed scheme in our method.

**Figure 5:**
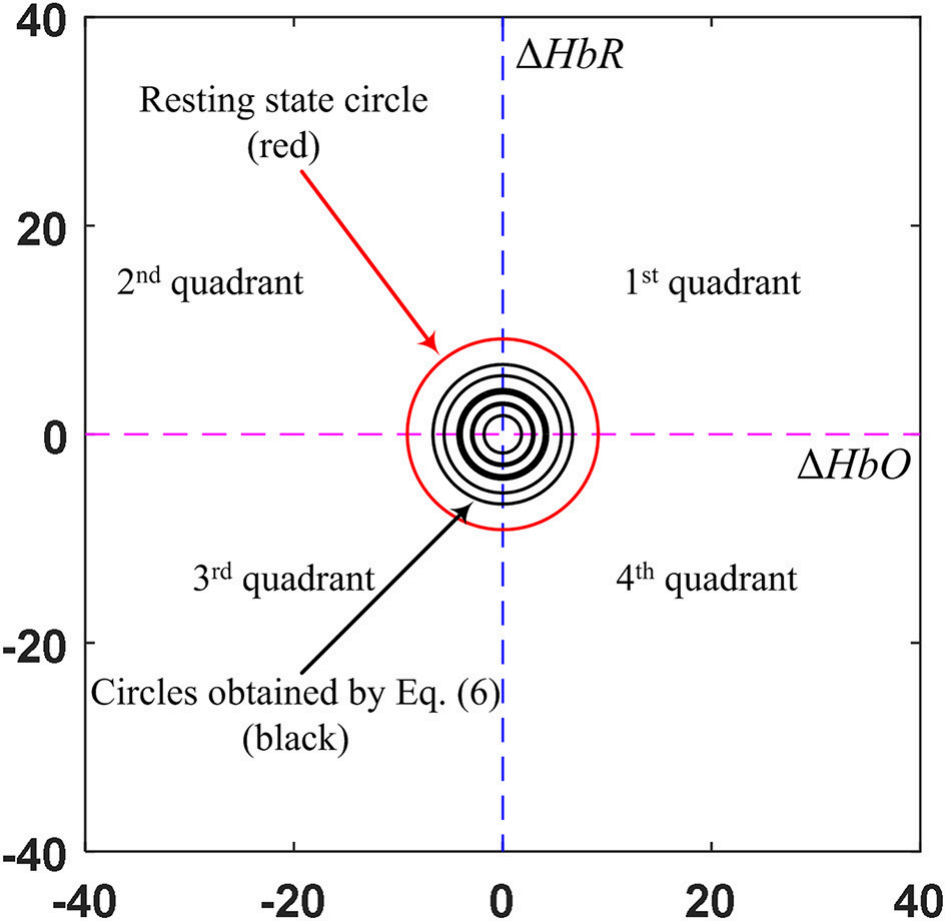
Comparison of the circles (in black) obtained by the EEG activated window upon trials and the resting state threshold circle (in red) (Sub. 3).

The black circle was made using the Δ*HbO* and Δ*HbR* values corresponding to the EEG activation window. In contrast, the red circle is the threshold circle made from the resting state, which uses the maximum values of Δ*HbO* and Δ*HbR*. Figure 5 shows an example of circles plotted based on the EEG activation window for Subject 3. The sizes of the black circles obtained in trials were different due to the differences in HbO and HbR values per trial. It is noted that the circle was made after *k* = 1 s after stimulation, because a moving window of 1 s was used to synchronize the EEG and fNIRS signals.

### Ideal trajectory for modified vector-phase analysis

**Figure 6:**
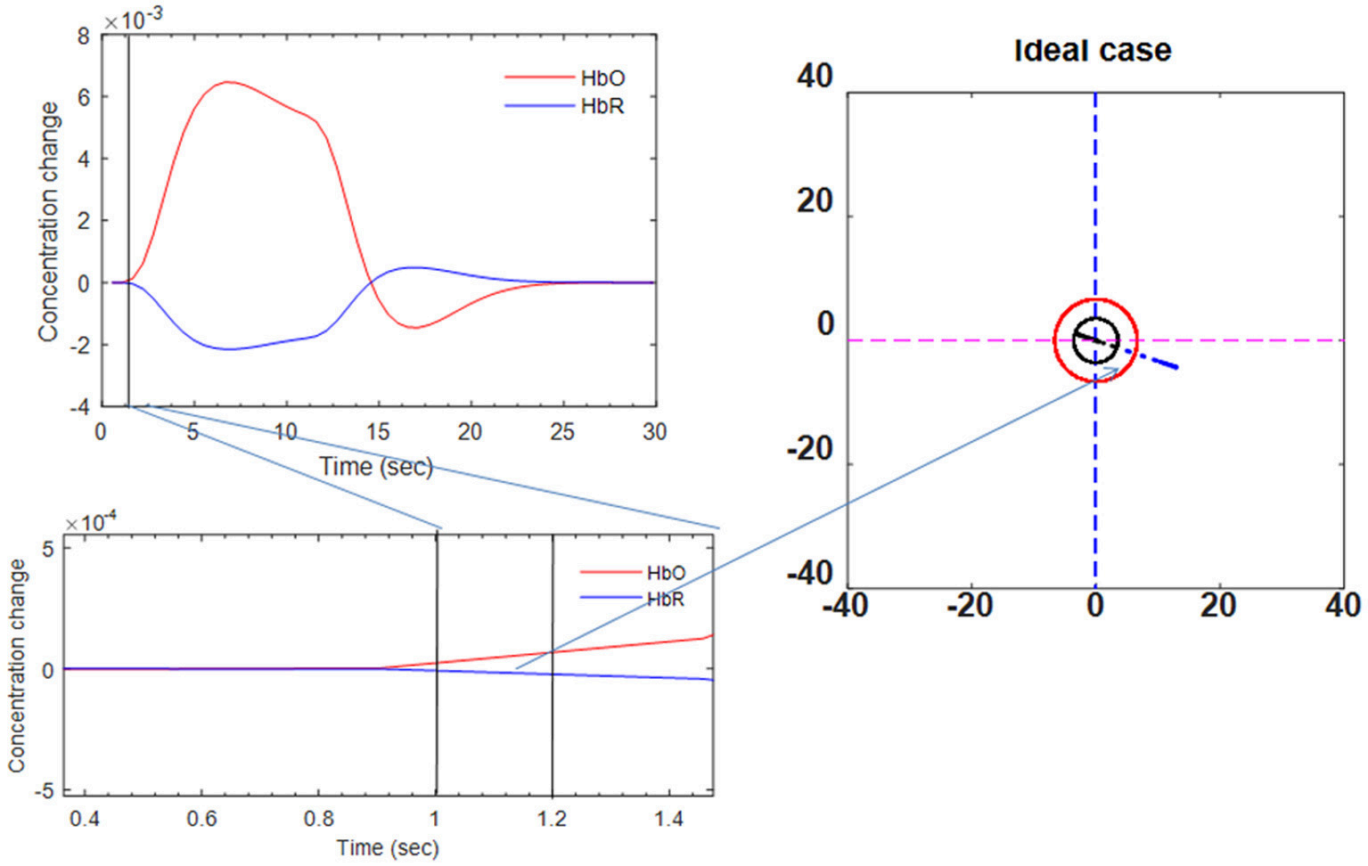
Ideal trajectory of HbO/HbR made by two-gamma-function model (see Phases 7 and 8 in Table 2).

A threshold circle (red solid circle) based on the maximum magnitude of the baseline was used for the detection of initial dips in previous studies (Hong and Naseer, 2016). We used a hemodynamic model based on two gamma functions (Δ*HbO* and Δ*HbR*) to estimate the ideal trajectory (Ye et al., 2009). A designed hemodynamic response function (dHRF) is defined as the convolution of the canonical hemodynamic response function (cHRF), *h*(*k*), and the stimulus, *s*(*k*), as follows:

(7) $$\begin{array}{l} u(k)=k_{1}\overset{k-1}{\underset{n=0}{\sum }}h\left(n\right)s\left(k-n\right), \end{array}$$

where *u* is the dHRF, *k*_1_ is the scaling parameter used to scale the amplitude of the response (*k*_1_ = 10 was used) if required, and *s*(*k*) is defined as

(8) $$\begin{array}{l} s\left(k\right)=\{\begin{array}{c} 1,\;\text{if }k\in task,\\ 0,\;\text{if }k\in rest, \end{array} \end{array}$$

where *rest* and *task* stand for the rest and the task periods, respectively. The cHRF, *h*(*k*), is generated as a linear combination of two gamma variant functions as follows:

(9) $$\begin{array}{l} h\left(k\right)=\alpha_{1}\left[\frac{{\left(k/\tau_{1}\right)}^{\left(\phi_{1}-1\right)}e^{-\left(k/\tau_{1}\right)}}{\tau_{1}\left(\phi_{1}-1\right)!}-\alpha_{2}\frac{{\left(k/\tau_{2}\right)}^{\left(\phi_{2}-1\right)}e^{-\left(k/\tau_{2}\right)}}{\tau_{2}\left(\phi_{2}-1\right)!}\right], \end{array}$$

where α_1_ is the amplitude, τ_*i*_ and ϕ_*i*_ (*i* = 1, 2) tune the shape and scaling, respectively, and α_2_ is the ratio of the response to the undershoot. If we had ideal data, the trajectory would be a straight line in the vector diagram. The ideal trajectory plot is shown in Figure 6.

## Results

In this paper, we propose a novel method to reduce the number of fNIRS signal detections by modifying the vector-phase analysis. A first threshold circle based on the magnitude of oxyhemoglobin (Δ*HbO*) and deoxyhemoglobin (Δ*HbR*) is placed in the vector diagram. A second circle is placed using the magnitudes of Δ*HbO* and Δ*HbR* corresponding to the window in which EEG is activated. A moving window of 1 s is used to synchronize and simultaneously decode EEG and fNIRS activities. The hemodynamic trajectory from the second circle to the first circle is estimated. Activity detection is performed if the trajectory moves from the first circle to the second circle in the fourth quadrant (where Δ*HbO* is positive and Δ*HbR* has a negative value). It takes around 0.5 s for the trajectory to move from the second circle to the first circle. Thus, a command can be generated in 1.5 s using the EEG activity detected within the 1 second moving window. To the best of the authors' knowledge, no method has been reported for the detection of fNIRS signals in 1.5 s.

**Figure 7:**
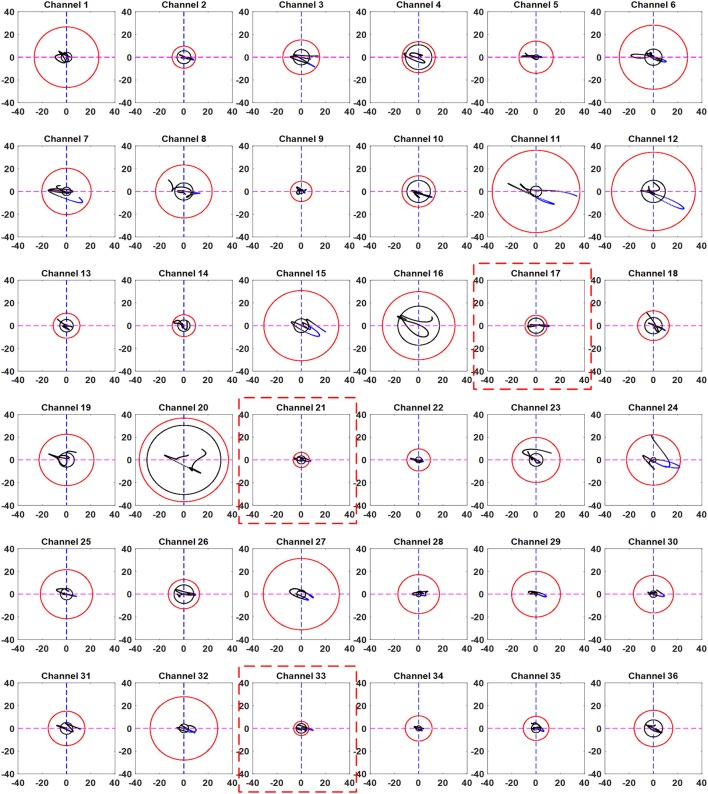
Trajectories of all the channels obtained by the proposed scheme (Sub. 3, Trial 3): The red dotted boxes show the channels in which the trajectory has crossed the EEG-based circle (black) in the fourth quadrant and moved away from the resting state threshold circle (red).

In addition, we used a moving window of 1 s on both EEG and fNIRS data to synchronize the windows. We measured the power of each window according to thumb tap activity. Threshold values of 15% over the baseline maximum value were used for the detected EEG activity. The threshold values were selected as a safety margin to avoid false detections. The results obtained using the proposed method for all channels with active channels highlighted for Subject 3 are shown in Figure 7. It can be seen from the figure that the trajectories of only a few channels went from the black circle to the red circle. Thus, the only channels that can be considered active are the ones in which the trajectory moved from the black circle to the red circle and crossed it. Thus, it is possible to identify the activated channels using the proposed method. In contrast with the selected channels shown in Table 3, only the channels that were activate were selected.

**Table 3:** Accuracies obtained using the proposed method.

**Subject**	**EEG+fNIRS accuracies using LDA (%)**	**Proposed EEG-fNIRS method (%)**
1	66.6	83.3
2	58.3	91.6
3	66.6	83.3
Mean	63.8	86.0

**Figure 8:**
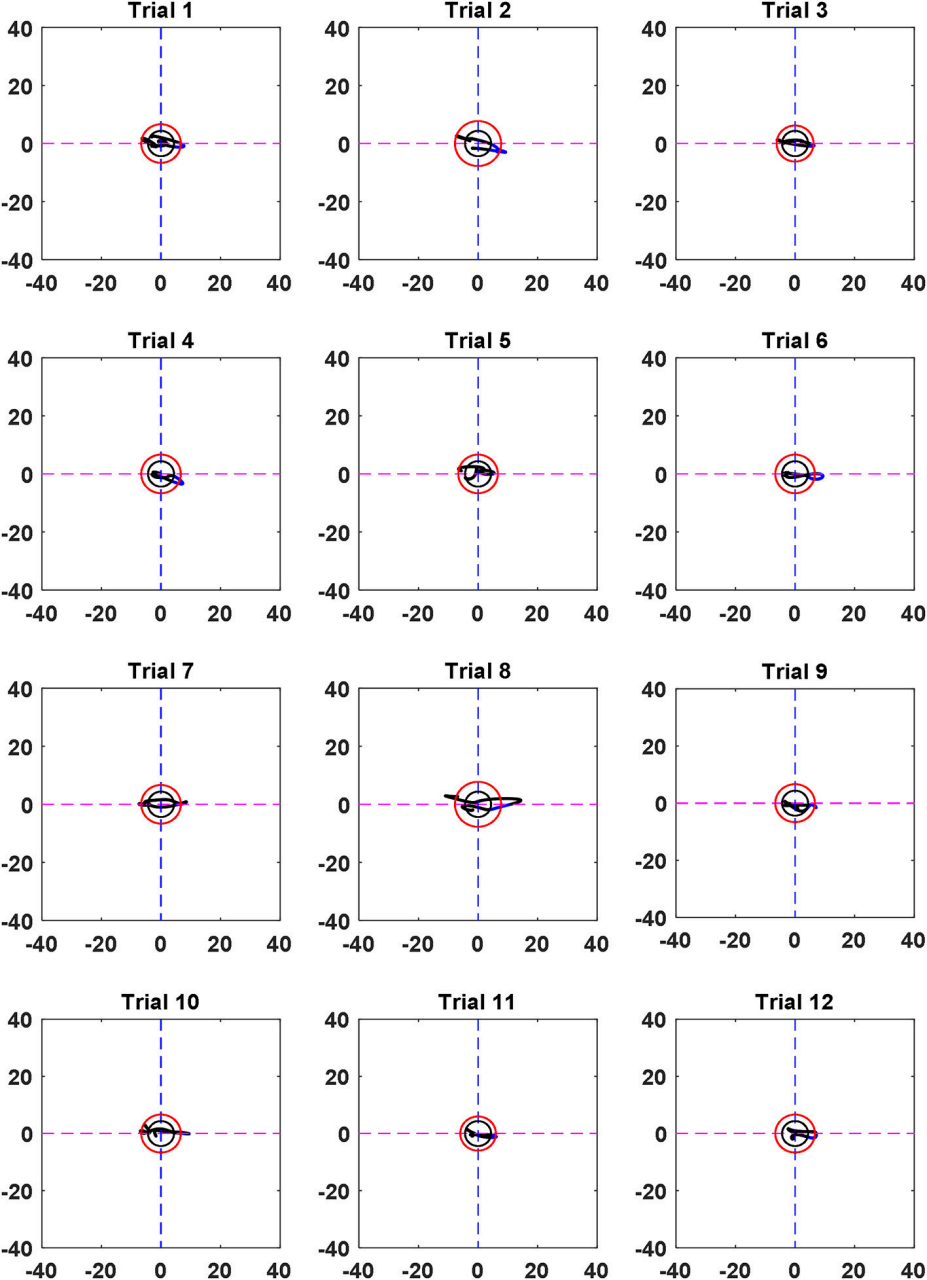
Trajectories of all 12 trials at Ch. 21 (Sub. 3).

Examples of the trajectories of all trials for Ch. 21 of Subject 3 are shown in Figure 8. It can be seen that the trajectories did not follow the same path in each trial. However, they moved from the smaller (black) circle to the larger (red) circle if the channel was active.

**Figure 9:**
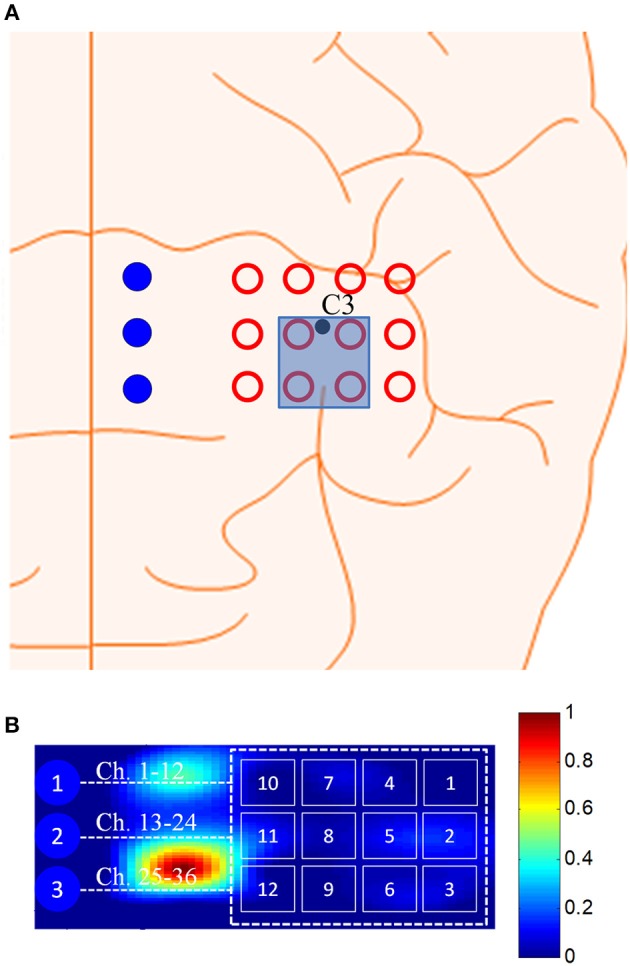
**(A)** Selected channels using the proposed method at *k* = 1.5 s, and **(B)** *t*-map at 10 s after stimulation (Sub. 3).

In fNIRS data analysis, the estimation of cortical activation and its localization are the most important steps. Cortical activation can be estimated by fitting the measured hemodynamic response (HR) to the predefined dHRF (Hu et al., 2010; Santosa et al., 2014), and its existence can be determined according to the *t*-values of the associated channels. The *t*-value is the ratio of the weighting coefficients resulting from the process of fitting the measured HR to the modeled dHRF and its standard error. A higher *t*-value means that the signal is highly correlated with the dHRF. Using the *t*-values, the regions of interest (ROIs), which consist of the channels in which the *t*-values are higher than the critical *t*-value (*t*_*crt*_) for the performed task, can be identified. In this study, *t*_*crt*_ was set to 1.65, according to the degrees of freedom (i.e., trial = 30 s, number of data points *N* = 30 × 9.19 = 275, *N* – 1 = 274) and the statistical significance level (i.e., 0.05 for the one-tailed test). The *t*-values were computed using the *robustfit* function available in MATLAB^TM^. The brain region that corresponds to the selected active channels is shown in Figure 9A. The channels were selected after 1.5 s of stimulation when the trajectory from the black circle touched the red baseline circle. Figure 9B shows the *t*-map for Subject 3 at *k* = 10 s after stimulation.

Finally, we also performed linear discriminant analysis (LDA) based classification to compare its results with the results of the proposed method. The EEG + ΔHbO features were estimated (as they have been reported to show the best results) using a window of 1.5 s. Table 3 shows a comparison of the accuracies obtained via the LDA-based method and via the proposed method. The results shows that accuracies achieved using the proposed scheme were higher in comparison with LDA-based classification.

## Discussion

One method for improving the classification accuracy by hybridizing EEG with fNIRS is based on the computation of probability scores (Fazli et al., 2012; Putze et al., 2014; Koo et al., 2015; Yin et al., 2015; Buccino et al., 2016). In this case, some decisions on command generation are made only if both modalities are active, while other decisions are made if only one of the two modalities is active. However, the problem with hybrid EEG-fNIRS is the temporal window used for simultaneous feature extraction. It takes around 6 s to compute the probability scores for fNIRS due to the inherent delay in the hemodynamic response, which is a long time for BCI-based control applications. This negates one of the advantages of EEG, that it can be used to detect a signal within a second. We minimized this problem with our method. A decision can be made by estimating the EEG window and by monitoring the fNIRS trajectory. If the trajectory goes from the smaller circle to the larger circle in the fourth quadrant, a decision on command generation can be made. Because the smaller circle is an indication that an activity is performed (as it will only be present if the EEG signal shows activation), the decision on activity detection can be made based on the direction of the trajectory. We plotted the trajectories from −2 to +3 s of stimulation (see Figure 7). The channel plots enclosed in a red dotted square are the ones in which the trajectory crossed the outer (red) circle, showing activation.

The optimal window proposed by a previous hybrid EEG-fNIRS study is 10 s (Tomita et al., 2014). That study showed significant improvement in the classification accuracy for a steady-state visual evoked potential task. However, the drawback was the time required for command generation. EEG signals can be detected within 1 s. We used the same time window (*k* = 1 s) to place a threshold circle using the maximum values of Δ*HbO* and Δ*HbR* (during this time window) and estimated the trajectory. The trajectory direction from the smaller circle to the larger circle indicates that the hemodynamic signal is increasing within a channel (see Table 2). It is important to have minimal motion artifacts within the baseline. An increased number of artifacts in the baseline would increase the size of R_1_ (red circle), and thus it would take longer to for the trajectory to reach the outer circle as the radius of R_1_ increases. If the distance between R_1_ and R_2_ is small, a decision on command generation can be made within 1.2 s. Therefore, it is most important to remove all noises in the baseline signal to further reduce activity detection time. In our case, we were able to detect the trajectory's motion from the smaller to the bigger circle in 0.5 s, and thus we were able to generate commands in 1.5 s. We further compared the accuracy obtained with the proposed method with that obtained via LDA classification with a 1.5 s window, for three subjects performing the thumb tapping task (see Table 3). We achieved an overall average accuracy of 86% with the proposed method, in contrast with that obtained via LDA (63.8% average accuracy). Our results show the feasibility of removal of the detected false EEG signals by hybridizing EEG with fNIRS using a 1.5 s window (for fNIRS) for BCI. Although there were variations in the hemodynamic responses on individual subjects due to trial-to-trial variability (Hu et al., 2013), the average classification accuracies obtained from the proposed method demonstrated its applicability for BCI purposes.

Another important issue is the sampling rates for EEG and fNIRS. In our case, we synchronized the EEG window (at 256 Hz) with the fNIRS window (at ~ 10 Hz). A high sampling rate for fNIRS can be used to describe the trajectory well. Additionally, an fNIRS system with a higher sampling rate can further minimize the signal detection time using the proposed method. Low sampling rates may not provide correct information on the trajectory from R_2_ to R_1_, as there would be few data points to estimate the hemodynamic response.

The advantage of the proposed method is the removal of false EEG signal detections using fNIRS to improve the classification accuracy of BCIs. In previous BCI schemes, different windows were used to simultaneously extract EEG features with fNIRS (Fazli et al., 2012; Blokland et al., 2014; Buccino et al., 2016). The probability scores for EEG and fNIRS were combined to generate a command. Instead of using conventional classifiers, our method is able to generate a command based on the trajectory crossing the threshold circles. Conventional classifiers require training data to make predictions on activity detection. However, our method updates the inner (black) circle radius with the changes in EEG power, and a decision can be made in real time without the need of training data to generate a command. Thus, further research on the proposed method would make it more effective than conventional classifiers.

Active channel identification is also another advantage of the proposed method. Activation maps were drawn at the end of the 10 s period based on the averaged HbO to see the activated brain regions. We found that the channels that showed high *t*-values in the 10 s *t*-map were the same as the ones identified “active” in the proposed method. Our results are consistent with the previous literature (Baker et al., 2014; Nguyen et al., 2016). It should be noted that the densely configured emitter-detector pairs in our study contained only 12 emitters and 3 detectors, resulting in only 36 channels to record brain activity in local brain regions in an area of 2 cm × 4.9 cm. However, if more emitter/detector combinations were available for forming more channels that covered a wider brain region, a more precise identification of active brain regions could be made. In addition, we focused only on the activation map generated by the average of all trials for a subject. The activation map can vary among individual subjects. Therefore, using an increased number of subjects with an increased number of trails could further narrow down the activated brain regions.

Our method can be extended for the simultaneous detection of initial dips along with the hemodynamic response. An initial dip is an early decrease in the hemodynamic response as result of neuronal firing. Recent fNIRS studies have reported using this signal in BCIs (Li et al., 2017; Zafar and Hong, 2017; Hong and Zafar, 2018). In our method, the decision on detecting hemodynamic signals was based on the trajectory in the fourth quadrant. The initial dip trajectory could be incorporated in the vector diagram to further enhance classification accuracy.

A limitation in this work is the small sample size of 3 participants. If the objective of this work were to find a new neuroscientific fact, a sufficient number of subjects should be utilized: The objective of this work was to establish a systematic method on how to combine EEG and fNIRS for brain signal detection from the same brain region. Using a small number of subjects in validating their work are also found in the literature: 2 subjects (Rakotomarnonjy and Guigue, 2008; Janani and Sasikala, 2017; Wyser et al., 2017), 3 subjects (Gratton et al., 1997; Obermaier et al., 2001; Edwards et al., 2013; Thanh Hai et al., 2013) and 4 subjects (Townsend et al., 2006) to name a few. For more details, a review paper of Fernández-Rodríguez et al. (2016) is referred. However, a new scientific (or cognitive) finding with the proposed method is highly desirable to utilize a large sample size in the future. Moreover, an accuracy comparison caused by gender-related effects can be performed in the future as well-since all subjects were male. In this study, the pre-processing of the data was done by only a band-pass filter. As the threshold circle (R_1_) in the vector phase analysis is highly dependent on the quality of the measured resting-state data, it is therefore highly recommended in the future to use any motion artifact correction algorithm prior to the selection of R_1_.

## Conclusion

In this paper, we have shown the feasibility of detecting an early fNIRS response using EEG as a marker. A modified vector-phase analysis was used as a classifier. Two threshold circles were placed in the vector diagram; one based on the maximum value of the resting state using Δ*HbO* and Δ*HbR* measurements, and the other using the window in which EEG activity was detected. The EEG and fNIRS data was synchronized using a moving window of 1 s. The detection of a decision activity occurred when the trajectory of the magnitudes of Δ*HbO* and Δ*HbR* crossed the active EEG-window-based threshold circle and touched the resting state circle. A command generation decision was made in 1.5 s on average. The proposed scheme showed potential for early hemodynamic response detection for BCIs.

## Author contributions

MJK has conducted the experiments, carried out the data processing, and wrote the first draft of the manuscript. UG participated in the data processing and revising the manuscript. K-SH has suggested the theoretical aspects of the current study, corrected the manuscript and supervised all the process from the beginning. All the authors have approved the final manuscript.

### Conflict of interest statement

The authors declare that the research was conducted in the absence of any commercial or financial relationships that could be construed as a potential conflict of interest.
